# Disruption of melatonin synthesis is associated with impaired 14-3-3 and miR-451 levels in patients with autism spectrum disorders

**DOI:** 10.1038/s41598-017-02152-x

**Published:** 2017-05-18

**Authors:** Cécile Pagan, Hany Goubran-Botros, Richard Delorme, Marion Benabou, Nathalie Lemière, Kerren Murray, Frédérique Amsellem, Jacques Callebert, Pauline Chaste, Stéphane Jamain, Fabien Fauchereau, Guillaume Huguet, Erik Maronde, Marion Leboyer, Jean-Marie Launay, Thomas Bourgeron

**Affiliations:** 1 Institut Pasteur, Human Genetics and Cognitive Functions Unit, Paris, France; 2 CNRS UMR 3571: Genes, Synapses and Cognition, Institut Pasteur, Paris, France; 3 Université Paris Diderot, Sorbonne Paris Cité, Human Genetics and Cognitive Functions, Paris, France; 4 Fondation FondaMental, 94000 Créteil, France; 5 Service de Biochimie et Biologie Moléculaire, INSERM U942, Hôpital Lariboisière, APHP, 75010 Paris, France; 6 University Paris Descartes, Sorbonne Paris Cité, 75005 Paris, France; 7 Child and Adolescent Psychiatry Department, Hôpital Robert-Debré, APHP, 75019 Paris, France; 8 INSERM U955, Psychiatrie Translationnelle, Université Paris-Est, 94000 Créteil, France; 9 Institute for Anatomy III, Goethe-Universität, 60590 Frankfurt/Main, Germany; 10 Département de psychiatrie, Hôpital Henri-Mondor – Albert Chenevier, APHP, Université Paris Est, 94000 Créteil, France; 11 Service Maladies Héréditaires du Métabolisme et Dépistage Néonatal, Centre de Biologie et de Pathologie Est, Hospices Civils de Lyon, 69500 Bron, France

## Abstract

Autism spectrum disorders (ASD) are characterized by a wide genetic and clinical heterogeneity. However, some biochemical impairments, including decreased melatonin (crucial for circadian regulation) and elevated platelet N-acetylserotonin (the precursor of melatonin) have been reported as very frequent features in individuals with ASD. To address the mechanisms of these dysfunctions, we investigated melatonin synthesis in post-mortem pineal glands - the main source of melatonin (9 patients and 22 controls) - and gut samples - the main source of serotonin (11 patients and 13 controls), and in blood platelets from 239 individuals with ASD, their first-degree relatives and 278 controls. Our results elucidate the enzymatic mechanism for melatonin deficit in ASD, involving a reduction of both enzyme activities contributing to melatonin synthesis (AANAT and ASMT), observed in the pineal gland as well as in gut and platelets of patients. Further investigations suggest new, post-translational (reduced levels of 14-3-3 proteins which regulate AANAT and ASMT activities) and post-transcriptional (increased levels of miR-451, targeting 14-3-3ζ) mechanisms to these impairments. This study thus gives insights into the pathophysiological pathways involved in ASD.

## Introduction

Autism spectrum disorders (ASD) are characterized by deficits in social communication and presence of stereotypy and restrictive patterns of interest. The genetic architecture of ASD is heterogeneous, shaped by a combination of hundreds of frequent and rare variants that can be different from one individual to another^1^. In this context of wide genetic heterogeneity, the identification of common biological features may reveal shared pathophysiological pathways. Several biological abnormalities have been reported in individuals with ASD, including neurochemical, immunological, endocrine or metabolic traits^2–4^. Among those, elevated blood serotonin is one of the most replicated findings^5–8^. A deficit in melatonin, from blood or urine of individuals with ASD, has also been described in several studies^8–13^, and is associated with increased peripheral N-acetylserotonin (NAS), the intermediate metabolite between serotonin and melatonin^8^.

Melatonin is a hormone mainly synthesized in the pineal gland during the night. It is a biological signal of light/dark cycles and is considered as a major circadian synchronizer^14, 15^. It is also a modulator of metabolism, immunity, reproduction and digestive functions^16^ and can directly modulate neuronal networks^17^. Melatonin appears as a therapeutic target of the frequently reported sleep disorders associated with ASD^18–20^. NAS also displays intrinsic biological properties: it is an agonist of the TrkB receptor and may thus share the neurotrophic properties of brain-derived neurotrophic factor (BDNF), the canonical TrkB ligand^21, 22^.

**Figure 1:**
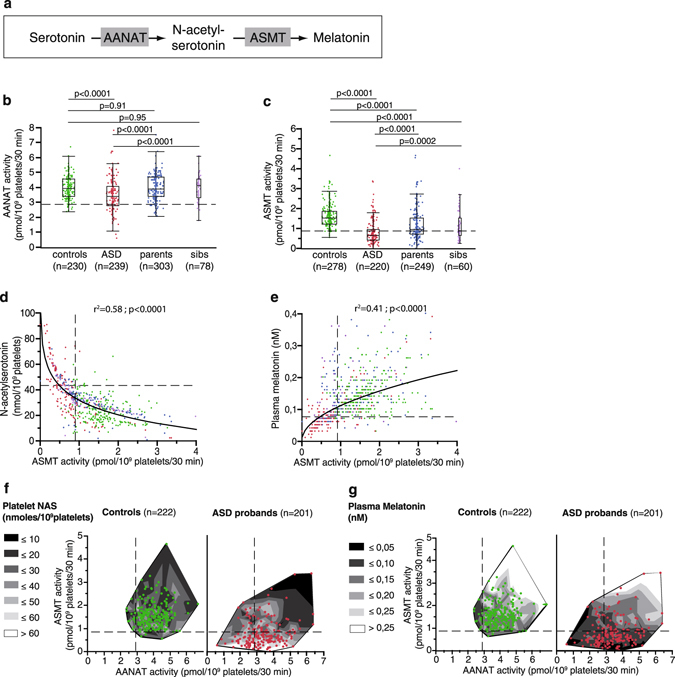
Exploration of the serotonin-melatonin pathway in the blood. **(a)** The serotonin and melatonin synthesis pathway. **(b)** Platelet AANAT activity. **(c)** Platelet ASMT activity. **(d)** Correlation between platelet NAS concentration and ASMT activity (regression after log transformation of ASMT, n = 579). **(e)** Correlation between plasma melatonin and platelet ASMT activity (regression after log transformation of melatonin and ASMT, n = 641). **(f)** Contour plot of platelet NAS concentration, AANAT and ASMT activities in patients (red dots) and controls (green dots). **(g)** Contour plot of plasma melatonin concentration, platelet AANAT and ASMT activities in patients (red dots) and controls (green dots). Blood samples were taken in the morning from 239 individuals with ASD, their first-degree relatives (303 parents and 78 unaffected sibs), and 278 controls. Boxes indicate medians and quartiles. Dashed lines indicate the threshold of the 95^th^ (for NAS) or 5^th^ (for melatonin, AANAT and ASMT) percentile of the control group. Groups were compared using the Wilcoxon two-sample test.

Serotonin conversion into melatonin involves two sequential enzymatic steps: N-acetylation of serotonin into N-acetylserotonin (NAS) by arylalkylamine N-acetyltransferase (AANAT, EC: 2.3.1.87), determining the timing of melatonin secretion, followed by methylation by acetylserotonin O-methyltransferase (ASMT, also called hydroxyindole O-methyltransferase HIOMT, EC: 2.1.1.4), determining the amount of melatonin secretion (Fig. 1a). The regulation of melatonin synthesis displays inter-species variability, including within mammals. In humans, it is considered to involve post-translational mechanisms, including AANAT phosphorylation, and stabilization of both AANAT and ASMT by formation of complexes with 14-3-3ζ proteins^23, 24^. In addition, 14-3-3ζ protein has been shown to be a target of miR-451^25–27^ which confers resistance to the deleterious effects of oxidative stress, which may play a role in the pathogenesis of ASD, promoting neuronal damage in genetically predisposed individuals^4^.

Based on ASMT assessment in blood platelets, we previously suggested that melatonin deficit in patients with ASD results from impaired melatonin synthesis^12^. Here, we investigated both AANAT and ASMT activities, not only in blood samples (platelets) from a large cohort of patients, their relatives and controls, but also in post-mortem tissues (pineal gland, the major source of melatonin, and gut [ileum samples], the major source of serotonin) from individuals with ASD and controls. We addressed the contribution of rare variants in *AANAT* and *ASMT* genes in ASD. Finally, we investigated 14-3-3 proteins in blood platelets and tissues, and miR-451 in plasma of patients with ASD.

## Results

### Disruption of AANAT and ASMT activities in blood platelets from individuals with ASD

We assessed ASMT enzyme activity in blood platelets from a large cohort of patients with ASD, their first-degree relatives and controls in order to achieve sample sizes of 239 patients, 303 parents, 78 unaffected siblings and 278 controls. Confirming previous results obtained on a smaller cohort^12^ (Supplementary Figure 1a–c), ASMT activity was significantly decreased in patients’ platelets compared to controls, and to a lesser extent in their first-degree relatives (Fig. 1c). A separate analysis of the two data sets is presented in Supplementary Figure 1d–e.

For the first time we also investigated AANAT activity in blood platelets. AANAT activity was significantly diminished in blood platelets from patients with ASD, but not in their relatives (Fig. 1b). These results assert a global disruption of melatonin synthesis in ASD, involving both AANAT and ASMT enzymes.

Platelet NAS and plasma melatonin were significantly correlated with ASMT activity in all status groups (Fig. 1d–e), with the strongest correlation observed for patients with ASD (NAS-ASMT regression after log transformation of ASMT: n = 193, r^2^ = 0.57, p < 0.0001; melatonin-ASMT linear regression: n = 201, r^2^ = 0.57, p < 0.0001). Accordingly, a profile analysis shows that in ASD patients a low plasma melatonin level and elevated platelet NAS content were both significantly associated with low platelet activities of both AANAT and ASMT (Supplementary Figure 2a). The contour plots (*i.e*. relationships between AANAT activity, ASMT activity and platelet NAS or plasma melatonin, and in ASD patients and controls) indicate ASMT activity as the most related to plasma melatonin and platelet NAS (Fig. 1f–g).

No correlation was observed between whole blood serotonin and either AANAT or ASMT activity (data not shown), indicating that the disruption of melatonin synthesis in platelets of patients with ASD is not sufficient to induce serotonin accumulation. This is consistent with the fact that melatonin synthesis represents only a small minority (about 1%) of serotonin catabolism.

Thus, taking as a threshold the 10^th^ percentile of the control group, a large proportion (74%) of ASD patients displayed a reduced platelet ASMT activity, associated with a decreased plasma melatonin and an increased platelet NAS content. Supplementary Figure 2b recapitulates the frequencies of the different biochemical profiles observed for patients with ASD: the most frequently observed profiles associated low plasma melatonin, low platelet ASMT activity, and high platelet NAS content (40% of ASD patients) with or without increased whole blood serotonin or decreased platelet AANAT activity. Interestingly, only 6% of ASD patients displayed a totally “normal” biochemical profile for the serotonin-melatonin pathway compared to 18% in the relatives and 70% in the controls. Finally, a significant association was observed between ASMT deficit and insomnia, as previously reported for melatonin deficit^8^. In contrast, no significant clinical correlates of these biochemical impairments were observed for core symptoms of ASD (Supplementary Table 1).

### Disruption of the serotonin-NAS-melatonin pathway in autopsy-derived pineal glands from individuals with ASD

**Figure 2:**
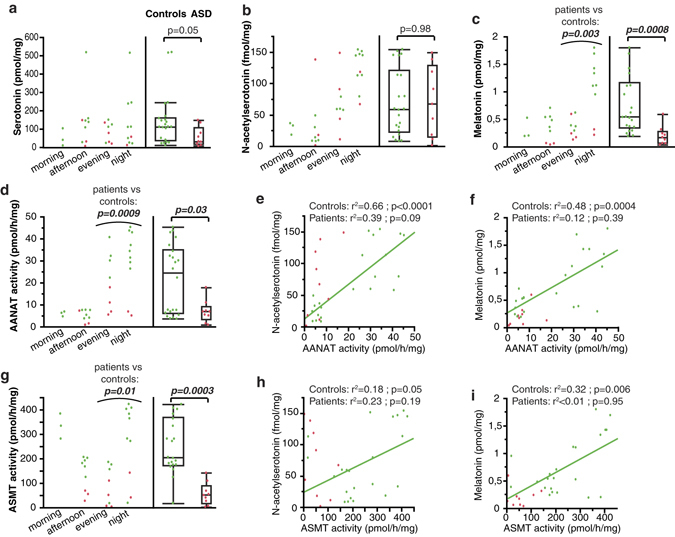
Characterization of the serotonin-melatonin pathway in autopsy-derived pineal gland samples from 9 individuals with ASD and 22 controls. **(a)** Serotonin content. **(b)** NAS content. **(c)** Melatonin content. **(d)** AANAT activity. **(e)** Correlation between NAS content and AANAT activity and regression line (green) for the control group (regression after log transformation of AANAT). **(f)** Correlation between melatonin content and AANAT activity, and regression line for the control group (green). **(g)** ASMT activity. **(h)** Correlation between NAS content and ASMT activity, and regression line for the control group (green). **(i)** Correlation between melatonin content and ASMT activity, and regression line for the control group (green). The ASD group was compared to the control group using Wilcoxon two-sample test. Boxes indicate medians and quartiles.

Considering that the regulation of the serotonin-NAS-melatonin pathway is likely tissue-specific and blood platelets are only a very minor source of melatonin, we assessed the complete serotonin-melatonin pathway (*i.e*. serotonin, NAS, melatonin, AANAT, and ASMT - Fig. 1a) in autopsy-derived pineal glands from 9 individuals with ASD and 22 controls. As melatonin synthesis is under strong circadian regulation in the pineal gland, analyses were stratified by time of death. Indeed, marked variations according to time of death were observed for melatonin (Fig. 2c, Kruskal-Wallis test: p = 0.009), NAS (Fig. 2b, p = 0.0005), AANAT (Fig. 2d, p = 0.003) and ASMT (Fig. 2g, p = 0.008), but not serotonin (Fig. 2a, p = 0.53).

Overall, melatonin (Fig. 2c), ASMT (Fig. 2g) and AANAT (Fig. 2d) were significantly decreased in individuals with ASD as compared to controls. Only two patients with ASD had nocturnal time of death, limiting the statistical power for nighttime analyses. Pooling evening and night tissue data reassert lower values for ASD patients *vs*. controls for AANAT (p = 0.0009), ASMT (p = 0.01) and melatonin (p = 0.03). Additionally, the melatonin and ASMT decrease remained significant even when considering individuals with diurnal (lights on) time of death. In control samples (but not in patients), the melatonin content of pineal glands displayed moderately strong, but significant correlations with both AANAT (Fig. 2f) and ASMT (Fig. 2i) activities.

NAS was not increased in the pineal glands of individuals with ASD (Fig. 2b), in contrast to our findings in blood platelets (Supplementary Figure 1b). The NAS content was correlated with AANAT and ASMT activities in control samples, but not in patients (Fig. 2e and h).

After correction for post mortem interval, no significant difference was observed for serotonin levels between individuals with ASD and controls (Fig. 2a).

Thus, although pineal glands do not reflect the peripheral increases of NAS and serotonin, they reassert the melatonin deficit observed in periphery, as well as a deficit in both ASMT and AANAT activities, confirming the impairment of melatonin synthesis in ASD.

### Disruption of the serotonin-NAS-melatonin pathway in autopsy-derived ilea from individuals with ASD

**Figure 3:**
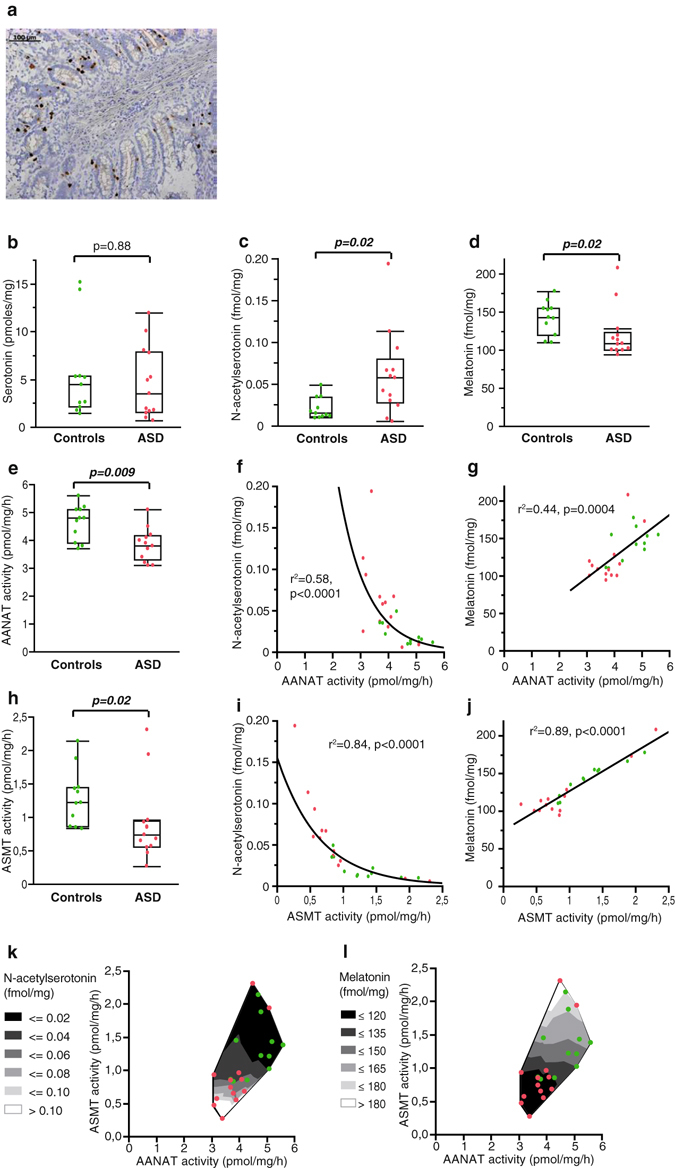
Characterization of the serotonin-melatonin pathway in autopsy-derived ileum samples from 13 individuals with ASD and 11 controls. **(a)** Example of chromogranin A immunostaining in one sample from a patient with ASD, assessing the presence of enterochromaffin cells. **(b)** Serotonin content. **(c)** NAS content. **(d)** Melatonin content. **(e)** AANAT activity. **(f)** Correlation between NAS content and AANAT activity (regression after log transformation of NAS). **(g)** Correlation between melatonin content and AANAT activity. **(h)** ASMT activity. **(i)** Correlation between NAS content and ASMT activity (regression after log transformation of NAS). **(j)** Correlation between melatonin content and ASMT activity. **(k)** Contour plot of NAS content, AANAT and ASMT activities in patient (red dots) and control (green dots) samples. **(l)** Contour plot of melatonin content, AANAT and ASMT activities in patients (red dots) and control (green dots) samples. The ASD group was compared to the control group using Wilcoxon two-sample test. Boxes indicate medians and quartiles.

The gastro-intestinal tract is the major source of serotonin, and another minor source of melatonin. Autopsy-derived ileum samples were obtained from 13 individuals with ASD and from 11 age- and sex-matched controls. Samples were investigated for the whole serotonin-melatonin pathway. Since tissue conservation is critical for autopsy-derived samples, and especially for gastro-intestinal tissues, the conservation state of samples and the presence of enterochromaffin cells (synthesizing serotonin and melatonin) were confirmed by immunohistochemistry using an anti-chromogranin A antibody (Fig. 3a). Enterochromaffin cells were detectable in all samples, including those displaying altered histological structures. In contrast to pineal glands, no effect of time of death was observed and the analyses were thus not stratified.

We observed no difference between individuals with ASD and controls for the serotonin content (Fig. 3b). In contrast, the melatonin content (Fig. 3d) of ileum samples as well as AANAT (Fig. 3e) and ASMT (Fig. 3h) activities were significantly decreased in patients with ASD compared with controls. Melatonin and AANAT activity were negatively correlated with post mortem interval, but the difference between individuals with ASD and controls remained significant after correction for post mortem interval (Wilcoxon two-sample test, p = 0.01 and 0.005 respectively). Although the melatonin level was significantly correlated with both AANAT (Fig. 3g) and ASMT (Fig. 3j) activities, the contour plots (Fig. 3l) indicated that ASMT activity was the most correlated factor to melatonin content in ileum samples from patients and controls. As in blood platelets (Supplementary Figure 1b), NAS was increased in ileum samples of individuals with ASD (Fig. 3c). A significant correlation was also observed between NAS, AANAT (Fig. 3f,k) and ASMT (Fig. 3i,k) activities.

Thus, the disruption of melatonin synthesis observed in the pineal gland of patients with ASD is also detectable in a peripheral tissue producing melatonin such as the gut, and, as in blood platelets, is associated with increased NAS. These results assert a global disruption of melatonin synthesis in ASD, involving both AANAT and ASMT enzymes, and affecting the pineal gland as well as peripheral tissues.

### Contribution of rare genetic variants in AANAT and ASMT in ASD

Considering that the biochemical impairment of melatonin synthesis observed in patients with ASD is shared by first-degree relatives (Fig. 1c, Supplementary Figure 1), we hypothesized that these impairments may be caused by rare mutations and hence sequenced *AANAT* and *ASMT* genes. The results were analyzed together with previously published sequencing data^12, 28–30^: taken together, 431 individuals with ASD and 365 controls were directly sequenced for *AANAT*, as well as 491 individuals with ASD and 490 controls for *ASMT*, respectively (Table 1). The functional impact of each mutation was determined *in silico* and *in vitro* after targeted mutagenesis. Overall, 4.7% of individuals with ASD and 3.9% of controls had a deleterious variant in one of the two genes encoding melatonin synthesis enzymes (p = 0.56). Taking as a threshold the 5^th^ percentile of the control group (0.88 and 2.9 pmol/10^9^ platelets/30 min for ASMT and AANAT, respectively), 2 patients with ASD were carrying ASMT deleterious variants (N17K and C273S) and displayed reduced platelet ASMT activity, while all patients carrying *AANAT* deleterious variants displayed normal platelet AANAT activity, suggesting that heterozygous *AANAT* mutations may not have a major impact *in vivo*, at least for peripheral, low-level enzyme expression (Supplementary Table 2).

**Table 1:** Mutation screening of AANAT and ASMT.

gene	**variant**	prediction in silico (polyphen2)	**activity in vitro**	**population**	**Controls**			**Controls**	**ASD**			**ASD**		total mutations	damaging mutations
				**reference**	Chaste 2011		this study	**Meta-analysis**			this study	**Meta-analysis**			
				**n** **= **	220		145	**365**			431	**431**			
*AANAT*	**T3M**	benign	reduced		0		0	**0**			4	**4**			
*AANAT*	**A13S**	benign	normal		0		0	**0**			1	**1**	**n(%) ASD**	**13(3.0%)**	**10(2.3%)**
*AANAT*	**R53C**	probably damaging	ND		0		0	**0**			1	**1**			
*AANAT*	**V62I**	benign	normal		1		0	**1**			1	**1**	**n(%) controls**	**8(2.2%)**	**7(1.9%)**
*AANAT*	**R71W**	probably damaging	ND		0		1	**1**			0	**0**	**OR (95% CI)**	**1.4(0.6–3.4)**	**1.2(0.5–3.2)**
*AANAT*	**T110M**	probably damaging	ND		0		0	**0**			1	**1**	**p**	**0.51**	**0.81**
*AANAT*	**A157V**	benign	normal		0		0	**0**			1	**1**			
*AANAT*	**A163V**	benign	reduced		3		2	**5**			4	**4**			
*AANAT*	**G177D**	probably damaging	reduced		1		0	**1**			0	**0**			
				**reference**	**Melke 2008**	**Pagan 2011**	**this study**	**Meta-analysis**	**Melke 2008**	**Johnson 2010**	**this study**	**Meta-analysis**			
				**n=**	255	**185a**	**50**	**490**	**250**	**109**	**132**	**491**			
*ASMT*	**L11F**	possibly damaging	reduced		0	0	0	**0**	0	0	1	**1**			
*ASMT*	**N17K**	possibly damaging	disrupted		0	0	0	**0**	1	0	1	**2**	**n(%) ASD**	**14(2.9%)**	**12(2.4%)**
*ASMT*	**V46M**	possibly damaging	ND		0	0	1	**1**	0	0	0	**0**			
*ASMT*	**E61Q**	possibly damaging	reduced		0	1	0	**1**	0	0	0	**0**	**n(%) controls**	**11(2.2%)**	**10(2.0%)**
*ASMT*	**K81E**	benign	normal		0	0	0	**0**	1	0	0	**1**	**OR (95% CI)**	**1.3(0.6–2.8)**	**1.2(0.5–2.8)**
*ASMT*	**splice site**	damaging	ND		0	0	1	**1**	2	1	0	**3**	**p**	**0.69**	**0.83**
*ASMT*	**M198R**	benign	ND		0	0	0	**0**	0	0	1	**1**			
*ASMT*	**Y201X**	probably damaging	ND		0	0	1	**1**	0	0	0	**0**			
*ASMT*	**D210G**	probably damaging	disrupted		1	0	0	**1**	0	0	0	**0**			
*ASMT*	**K219R**	benign	normal		1	0	0	**1**	0	0	0	**0**			
*ASMT*	**P243L**	probably damaging	reduced		1	0	0	**1**	0	0	0	**0**			
*ASMT*	**I269M**	possibly damaging	reduced		0	0	0	**0**	0	0	1	**1**			
*ASMT*	**C273S**	probably damaging	reduced		1	0	0	**1**	0	0	1	**1**			
*ASMT*	**G278A**	possibly damaging	reduced		0	0	0	**0**	1	0	0	**1**			
*ASMT*	**R291Q**	probably damaging	disrupted		1	0	0	**1**	0	0	0	**0**			
*ASMT*	**L298F**	possibly damaging	disrupted		2	0	0	**2**	2	0	0	**2**			
*ASMT*	**H318D**	benign	reduced		0	0	0	**0**	0	0	1	**1**			
													**% ASD**	**5.9%**	**4.7%**
										**total (ASMT+AANAT)**			**% controls**	**4.4%**	**3.9%**
													**OR (95% CI)**	**1.3 (0.7–2.4)**	**1.2 (0.6–2.3)**
													**p**	**0.37**	**0.56**

The missense, nonsense and splice-site variants identified in this study and in previously published sequencing studies are indicated. The frequencies of mutations observed in individuals with ASD were compared with those observed in the control groups using Fisher’s exact test.

aThe total sample size published^30^ was n = 440 controls, and included the 255 controls previously described^12^.

We also sequenced all ASMT exons of 7 patients and 17 controls that we assessed for enzyme activity in the pineal gland. For *ASMT*, we identified 2 previously identified rare non synonymous variants: K81E in a patient and E288D in a control. However, we have previously shown, using functional assays and *in vitro* mutagenesis, that these variants do not affect ASMT activity^31^.

Finally, we genotyped a recurrent *ASMT* microduplication (spanning exon 1 to 7), identified in 12 out of 290 individuals with ASD (4.1%) and in 10 out of 324 controls (3.1%) (OR[CI_0.95_] = 1.4[0.6–3.2]; Pearson’s chi-square: p = 0.48). Thus, using this PCR approach, we do not confirm the enrichment of microduplication of *ASMT* in individuals with ASD^32^. Among patients carrying the microduplication and for whom biochemical and clinical data were available, 5 out of 8 displayed reduced platelet ASMT activity (Supplementary Table 2).

In our previous study^8^, we reported an association between melatonin deficit and sleep disorders in patients with ASD. Among patients carrying rare variants and for whom sleep information was available, one out of 4 patients with an *ASMT* deleterious variant, 2 out of 4 with a deleterious *AANAT* variant, and 5 out of 8 patients with the *ASMT* microduplication reported insomnia and/or sleep phase delay (Supplementary Table 2).

### Reduction of 14-3-3 proteins and increase of miR-451 in samples from patients with ASD

**Figure 4:**
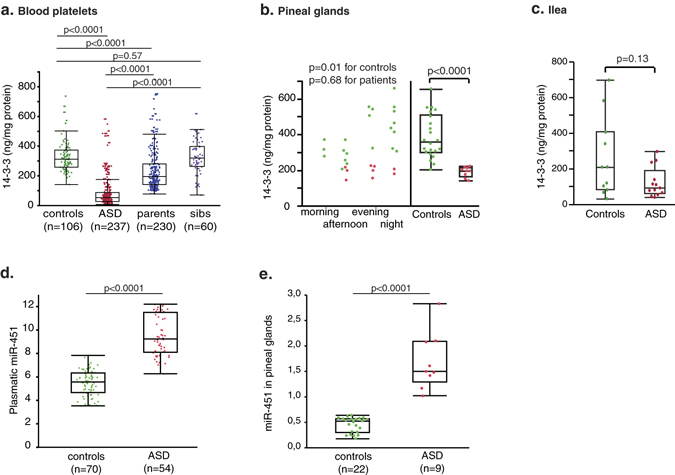
Amounts of 14-3-3 proteins and miR-451 in patient (red dots) and control (green dots) samples. **(a)** 14-3-3 proteins in blood platelets. **(b)** 14-3-3 proteins in autopsy-derived pineal glands. **(c)** 14-3-3 proteins in autopsy-derived ileum samples. **(d)** miR-451in plasma. **(e)** miR-451 in pineal glands.

The low frequency of rare variants in *AANAT* and *ASMT* genes in patients with ASD is not sufficient to explain the high frequency of melatonin deficit observed in these patients. In a previous study^8^, we proposed that post-translational regulations of AANAT and ASMT activities, involving the formation of complexes with 14-3-3 proteins^23, 24^, may be impaired in ASD, and reported low 14-3-3 levels in a small platelet sample from ASD patients. Here, we extended these preliminary findings to larger groups of controls (n = 106 *vs*. 70), patients (n = 237 *vs*. 40) and their relatives, confirming a strong reduction of 14-3-3 in platelets of patients with ASD, and revealing intermediate levels in parents (Fig. 4a), as for melatonin, NAS and ASMT (Fig. 1c, Supplementary Figure 1). Furthermore, we measured 14-3-3 in pineal glands from patients with ASD and found that, like AANAT and ASMT activities, it displays significant variations depending on time of death in controls, and is markedly reduced in samples from patients (Fig. 4b). In ileum, 14-3-3 tended to be reduced in patient samples, although this trend did not reach significance, possibly because of a lack of statistical power (Fig. 4c). These findings strengthen our hypothesis of a melatonin deficit resulting from altered 14-3-3-dependent post-translational regulations of AANAT and ASMT activities in ASD. In addition to an immunofluorescent co-localization of the 14-3-3 and the ASMT proteins with p31T-AANAT in human pinealocytes^24^, we found evidence that 14-3-3 proteins are able to regulate ASMT activity: firstly the pineal ASMT activity of 3 months 14-3-3ζ ko mice on a SV129 background^33^ was only 60.8 ± 3.8% (n = 8; 4 females, 4 males) as compared to control mice; secondly preincubation with difopein (a blocking agent of 14-3-3-target interactions)^34^ 1 µM for 1 h resulted in a 43.1 ± 4.6% (n = 4) reduction of the activity of a semi-purified bovine ASMT preparation (the one used for the radioenzymatic determination of 5-HT)^35^.

Since 14-3-3ζ is one of the isoforms most implicated in the regulation of melatonin synthesis^23, 24, 36^ and since 14-3-3ζ was reported to be repressed by miR-451^25–27^, we measured this micro-RNA in plasma and pineals. Plasmatic and pineal miR-451 were significantly increased in patients with ASD compared to controls (Fig. 4d and e), thus suggesting a post-transcriptional epigenetic mechanism for 14-3-3 impairment.

## Discussion

This study investigates for the first time melatonin synthesis in the pineal gland and in the gut from patients with ASD. The pineal gland is the only source of elevated nocturnal melatonin with levels compatible with most roles attributed to this hormone. Our findings reinforce the hypothesis that the nocturnal increase in circulating melatonin is reduced in patients with ASD. The results reveal that this melatonin deficit is caused by enzymatic disruption of both AANAT and ASMT, the two enzymes involved in melatonin synthesis. These impairments were observed in the pineal gland as well as in gut and blood platelets. In these two latter tissues, ASMT appeared as the limiting factor of melatonin synthesis, both in controls and ASD patients. In contrast, the regulation of melatonin level appeared to be more complex in the pineal gland, where the activities of both enzymes determine the melatonin level. This is consistent with the fact that, unlike in gut and platelets, ASMT activity was much higher than AANAT activity in pineal glands. Furthermore, AANAT and ASMT activities are cycling in the pineal gland and alternate as the limiting factor of melatonin synthesis according to the time of the daily cycle^37^.

Since melatonin secretion is known to be synchronized with light exposure, it may be speculated if the melatonin deficit observed in plasma or in the pineal gland is part of the pathophysiology of ASD, or rather a consequence of environmental bias resulting from ASD condition, e.g. insomnia associated with increased light exposure at night. However, tissue or blood samples were not collected in controlled light conditions. The pineal gland receives environmental cues from the retino-hypothalamic tract, and could be affected by such environmental bias. In contrast, melatonin synthesis in peripheral tissues such as blood platelets and gastro-intestinal tract is not directly regulated by light exposure: thus, the consistent findings of impaired melatonin synthesis in pineal glands, but also in peripheral tissues of patients with ASD, are not suggestive of a secondary or artefactual phenomenon.

One limitation to the tissue study is the small number of patient samples, limiting statistical power, especially in the pineal gland where stratification according to time of death is needed. In particular, only two patients with pineal gland samples had nocturnal time of death. However, in spite of this limitation, melatonin and ASMT decrease remained significant even for patients with diurnal time of death, when ASMT activity and melatonin secretion are at lowest level and the sensitivity to detect impairment in this pathway is expected to be lower. This is consistent with the significant decrease of plasmatic melatonin observed in patients with ASD even when sampled in the morning^8^.

In gut and platelets, the reduction of ASMT activity observed in patients with ASD appeared to be correlated with an accumulation of NAS, the intermediate metabolite between serotonin and melatonin. This increase of NAS was not observed in the pineal gland, and this may also result from the different stoichiometry of the two enzymes in this tissue compared with “peripheral” sources. Indeed, in contrast with data from platelets and ileum, pineal NAS was not correlated with ASMT activity, but with AANAT activity, although with a complex pattern as observed for melatonin. In the pineal glands of ASD patients, NAS levels may be determined by its degradation (by ASMT) and its synthesis (by AANAT), both being impaired. In platelets, unlike melatonin which is a diffusible molecule, NAS is co-stored with 5-HT in dense granules and its release is controlled^38^. By contrast, using high concentrations (10 mM) of indoleamines, it has been reported that melatonin and NAS are highly permeant molecules that will leak out of a cellular model for pinealocytes as rapidly as they are synthesized (ratio of estimated extracellular concentrations for melatonin, NAS, and 5-HT: 1:0.18:0.006)^39^. Whether or not NAS is accumulated in the brain of patients with ASD, where it may exert its proper biological functions, and especially its neurotrophic effect^21, 22^, is still to be determined.

The exact mechanisms of the disruption of AANAT and ASMT activities in ASD patients remain to be elucidated. Considering the familial aggregation of these biochemical traits, we considered a genetic hypothesis and screened *AANAT* and *ASMT* genes for rare variants. However, although rare variants may explain melatonin deficit in a subset of families, their low frequency was far from sufficient to explain the high rates of biochemical impairments reported in patients, and we found no significant enrichment of these variants in the population of patients with ASD compared with controls. Some functional polymorphisms in the *ASMT* promoter (rs4446909 and rs5989681) may have a role as they have been associated with the risk of ASD^12^. This association finding was not fully replicated in independent studies, although a similar trend was observed^29, 40, 41^. Interestingly, the same SNPs have also been associated with bipolar disorder^42, 43^, major depressive disorder^44^, and depressive symptoms in individuals with sleep phase delay^45^.

As an alternative to a defect in the genes coding for AANAT and ASMT, we propose that the abnormal reduction of 14-3-3 proteins^26^, first identified as activators of tryptophan and tyrosine hydroxylases^46^, could lead to a defect of AANAT^23, 36^ and ASMT activities^24, present study^, and ultimately to a melatonin deficit in ASD. Indeed, the 14-3-3 protein level was decreased in all three materials investigated in patients compared to controls. This post-translational hypothesis is consistent with the impairments of both AANAT and ASMT and, as suggested by exome sequencing in multiplex autism families^47^, polymorphisms in *YWHA* genes involved in 14-3-3 protein synthesis, already reported for ADHD, schizophrenia, and bipolar disorder^48^, are presently investigated in our lab. Finally, the platelet and pineal 14-3-3 decrease was found to be associated with an increase of plasma and pineal miR-451, reasserting in ASD the regulatory role of miR-451 towards 14-3-3^25–27^.

The roles of 14-3-3, miR-451 and melatonin deficits in the etiologic architecture of ASD remain to be elucidated. ASD are complex, heterogeneous and multifactorial diseases^1, 49, 50^. The high frequency of melatonin deficit in patients^8^ (Supplementary Figure 2b) is particularly striking within this paradigm, and might suggest that 14-3-3/melatonin impairments are part of the core pathological processes in ASD. In a multifactorial paradigm, a 14-3-3/melatonin deficit may be a susceptibility factor which, in conjunction with genetic variants in ASD-associated genes, would result in a pathological phenotype. The observation of melatonin impairments in parents of patients with ASD can fit with this hypothesis. Beyond the hundreds of reported susceptibility genes and loci in ASD, functional convergences have been identified to common pathways, including synaptic function, neuronal development and signaling, or chromatin remodeling. Non-genetically determined impairments, such as immune dysfunction, have also been implicated^49^. Alternatively, a 14-3-3/melatonin deficit may be a common phenomenon secondary to the disruption of one or several of these processes. In this case, considering the pleiotropic functions of the hormone melatonin and of the regulatory 14-3-3 proteins, it is likely that 14-3-3 and melatonin deficits would in turn exacerbate neuronal dysfunction(s). These findings provide support for future exploration into the potential role of the melatonin synthesis pathway in the treatment of ASD.

In conclusion, this study provides support for a frequent melatonin synthesis defect in ASD and reports for the first time that this abnormal melatonin reduction is caused by deficits of both AANAT and ASMT activities in pineal glands, gut, and blood platelets of patients. These results also open new perspectives on the post-translational and post-transcriptional mechanisms involved and provide insights into the common pathways of autism pathophysiology.

## Patients and Methods

### Methods were performed in accordance with the relevant guidelines and regulations

#### Autopsy-derived tissue samples

Ileum and pineal gland samples from individuals with ASD and controls were obtained from the NICHD Brain and Tissue Bank for Developmental Disorders of the University of Maryland (Baltimore, MD, USA), the Autism Tissue Program (Autism Speaks, Princeton, NJ, USA) and from the Anatomy Department of the Goethe Universität (Frankfurt, Germany). Written informed consents were obtained from the donors’ next of kin. Samples were frozen after autopsy and kept at −80 °C. Groups’ characteristics are described in Supplementary Table 3. Biochemical analyses were performed after tissue disruption and homogenization. The impact of post mortem interval (PMI) was assessed by linear regression (parameter measured = f[PMI]) in order to take into account the post mortem stability of the parameter investigated. For parameters displaying correlation with PMI, a correction was applied as follows: parameter corrected = parameter measured − (correlation coefficient × PMI). All figures present the raw data. Analyses performed with corrected data are described in the main text. Despite the fact that AANAT is known to display a very short half-life (a few minutes) in rodents^15^, there are major differences in its regulation among species. AANAT and ASMT activities have previously been shown to be stable when measured in human pineal organs held under the usual post-mortem conditions^51^, and no significant correlation was observed here between AANAT and ASMT activities and PMI.

#### Subjects, clinical evaluations, blood samples

The clinical evaluations and blood sampling of patients with ASD (diagnosed according to DSM-IV), their relatives, and control subjects investigated for genetics and blood biochemistry have been detailed previously^8^. The local Institutional Review Boards (Comité de Protection des Personnes Ile de France IX) approved this study. Written informed consents were obtained after oral and written information from all participants of the study, and from the children’s parents when subjects were under 18.

#### Biochemical measurements

Melatonin in plasma and tissues was measured using a radioimmunoassay (RK-MEL, Bühlmann, Switzerland) according to the manufacturer’s instructions. Serotonin was measured by high-performance liquid chromatography^52^. NAS, as well as enzyme activities of AANAT, ASMT were determined by radioenzymology. Technical details of enzymatic assays and circulating miR-451 determination are included as supplementary information.

The amount of 14-3-3 proteins was determined using the commercial 14-3-3 Pro ELISA kit from MyBioSource (San Diego, CA, USA). The immunogen used to produce the antibodies is recombinant full length human 14-3-3 γ. The kit has about 40% cross reactivity with 14-3-3 ζ, ε, σ, τ (MyBioSouce, personal communication).

#### Sequencing of AANAT and ASMT and genotyping of ASMT microduplication

*AANAT* (the 3 coding exons) and *ASMT* (promoter B and the 9 coding exons) were sequenced according to the previously published protocols^12, 28^. Technical details are included in Supplementary Information. Mapping and genotyping of ASMT microduplication are detailed in Supplementary Table 5.

#### Statistical analyses

Statistical analyses were performed using JMP Pro 10 (SAS, Toronto, ON, Canada) and Stata 11.0 (StataCorp LP, College Station, TX, USA). Since most biochemical parameters were not normally distributed, categorical and non-parametric statistical tests were preferred. Error type I was chosen at 0.05. The statistical tests used for this study were Wilcoxon two-sample test, Kruskall-Wallis test, Pearson’s chi-square test, Fisher’s exact test, and linear regression.

## Electronic supplementary material

Supplementary information
