# Supplementary material for: Disruption of melatonin synthesis is associated with impaired 14-3-3 and miR-451 levels in patients with autism spectrum disorders
Source: Sci Rep. 2017 May 18;7:2096. doi: 10.1038/s41598-017-02152-x (PMC5437096; doi:10.1038/s41598-017-02152-x)
Supplement: Supplementary file 1 — Supplementary information [file 41598_2017_2152_MOESM1_ESM.pdf]

Disruption of melatonin synthesis is associated with impaired 14-3-3 and miR-451 levels in patients with autism spectrum disorders

Cécile Pagan, Hany Goubran-Botros, Richard Delorme, Marion Benabou, Nathalie Lemière, Kerren Murray, Frédérique Amsellem, Jacques Callebert, Pauline Chaste, Stéphane Jamain, Fabien Fauchereau, Guillaume Huguet, Erik Maronde, Marion Leboyer, Jean-Marie Launay & Thomas Bourgeron.

Supplementary information

Clinical evaluations

All individuals, including controls, were enrolled after a medical and psychiatric assessment using the Diagnostic Interview for Genetic Studies for adults and Kiddie-Schedule for Affective Disorders and Schizophrenia (K-SADS) for children. Probands with a total score >30 at the Social Responsiveness Scale (SRS)[1] or for which a diagnosis of ASD was suspected, underwent additional screening with the Autism Diagnostic Interview-Revised with a parent[2] and/or the Autism Diagnostic Observation Schedule[3]. The final diagnosis of ASD was made when clinical assessment diagnosis coincided with algorithm-suggested diagnosis of autism on either or both of the Autism Diagnostic Interview-Revised and the Autism Diagnostic Observation Schedule. Repetitive behaviors were assessed using the RBS (Repetitive Behavior Scale)[4]. General medical history (including neurological, gastro-enterological and other conditions) was checked during interviews. For probands with ASD, a cognitive level was assigned using appropriate tests (Wechsler scales or Raven's progressive matrices for nonverbal individuals). Intellectual disability was defined as verbal and performance IQ < 70. Sleep difficulties were assessed in probands by self-report (and/or parent questionnaire for probands who were themselves not sufficiently verbal to describe sleep behaviors) during interviews. Standard karyotyping, fragile X testing, MRI and electroencephalography were performed whenever possible. Individuals diagnosed with medical disorders related to ASD, such as fragile X syndrome, Rett syndrome or tuberous sclerosis, were excluded from the study.

Methods for biochemical measurements

Platelet or tissue lysis was achieved by -SH-activated toxin treatment[5]. 150 hemolytic units of alveolysin purified to homogeneity (equivalent to about 375 ng or 6 pmoles of protein) were added (10:1) to platelet suspensions or tissue homogenates at 4°C and the suspensions warmed to 37°C for enzyme measurement. This amount of toxin binds to platelets or cell fragments at 4°C and elicits complete lysis at 37°C, since about 16 molecules of toxin are sufficient to lyse one human platelet[6]. Separate experiments showed that the addition of equivalent amounts of toxin to cells already disrupted by homogenization produced no change in the rates of enzyme activities measured at least in duplicate. Under the conditions employed here, enzyme rates were linear with time and with platelet or cell protein concentrations ranging from 0.01 to 0.1 mg of protein per mL.

Measurement of AANAT activity[7] was performed at 37°C in 1.5 mL polypropylene microtubes. The reaction was started by the addition of enzyme preparation (25 µL) to 40 µL buffer containing 20 mM sodium citrate (pH 7.00), 300 mM NaCl, 1 mM EDTA, 0.05 mg/ml BSA, 50 nM [acetyl-^{3}H]-CoA, and 500 nM tryptamine. After 20 min the reaction was stopped by adding 1 mL of chloroform and vortexing the mixture. The reaction product, N-[^{3}H]-acetyl tryptamine, was extracted into the chloroform which was then washed once with 0.1 mL of 0.1 mM sodium phosphate buffer, pH 6.80. A 500 µL aliquot of the chloroform was then transferred to a scintillation vial. Three mL of scintillation fluid were added and the radioactivity was measured by a liquid scintillation counter (Beckman LS 6000SC).

Reaction mixtures for ASMT assay[8] contained in a final volume of 50 µL the following: N-acetylserotonin (1 mM), tissue homogenate (25 µL), and 0.1 mM S-[methyl-^{3}H]-adenosyl-L-methionine. Reactions were carried out in plastic Eppendorf tubes at 37°C for 30 min in 50 mM sodium phosphate buffer (pH 7.90). Reactions were stopped by addition of 100 µL of 0.5 M borate buffer (pH 10.50) to the tubes. Blanks consisted of reaction mixtures minus N-

acetylserotonin. One mL of chloroform was added. The mixture was vortexed twice for 15 s to extract the product, [^{3}H]-melatonin, into the chloroform. The organic layer was washed once with borate buffer, and an aliquot (500 µL) was dried and its radioactivity measured.

In order to verify that the activities measured in platelets reflect authentic AANAT and ASMT and not non-specific actions of other N-acetyltransferases and methyltransferases, si-RNA specific for each enzyme (SR300008 and SR300317, Origene; 1 nM final concentrations after transfection by the siTran 1.0 as recommended by Origene) were used. Again the SH-activated toxin treatment of platelets allowed complete enzyme inhibition within 4 hours: the obtained values (n = 5) were similar (AANAT: 158 ± 24 dpm) or below (ASMT: 522 ± 342 dpm) the reagent "blank" values (AANAT: 164 ± 13 dpm; ASMT: 987 ± 213 dpm); t.e corresponding assay values were 3660 ± 410 dpm (AANAT) and 4804 ± 1032 dpm (ASMT).

Total RNA was extracted from plasma samples (haemolysed samples were avoided since haemolysis was reported to alter miR plasma content[9]) and pineal glands using the mirVana PARIS kit (Ambion, Thermo Fisher Scientific, St-Quentin en Yvelines, France). Spiked-in synthetic *C. elegans* miRNA controls (cel-miR39, 54 and 238; Qiagen, Courtaboeuf, France) were added to samples for correction of extraction efficiency. After DNAse treatment, RNAs were reverse transcribed with the miScript reverse transcription kit (Qiagen). cDNA was diluted 10-fold before quantitative PCR with the miScript SYBR Green PCR kit (Qiagen). A control without reverse transcriptase and a control without RNA were added to each PCR plate to ensure the absence of contaminating DNA and to check for non-specific amplification, respectively. Expression values were normalized using the mean Ct of the spiked-in controls and calculated with the $2^{-\Delta Ct}$ formula. Additional technical details have previously been published[10].

Sequencing of *AANAT* and *ASMT*

Primers and PCR conditions for exon amplification and sequencing are listed in Supplementary Table 4. Non-synonymous mutations were confirmed by sequencing an independent PCR product. The segregation of mutations within families was determined when DNA was available for relatives. The nomenclature of genetic variations was determined according to the following reference protein sequences in Ensembl database: ENSP00000370627 (345 amino acids) for ASMT, and ENSP00000376282 (207 amino acids) for AANAT. Exon 6 of *ASMT* is a LINE insertion and yields an inactive form of the protein, thus rare variants located in exon 6 were not considered as coding mutations.

The functional impact of each non-synonymous coding mutation was assessed both *in silico* using Polyphen2 algorithm (http://genetics.bwh.harvard.edu/pph2/) and *in vitro* by targeted mutagenesis, as described previously[11]. A pool-analysis of coding mutation frequencies was performed including our results and studies published and referenced in PubMed, in which a direct sequencing of all exons of *ASMT* or *AANAT* was performed in individuals with ASD and/or in controls.

Supplementary Table 1: Clinical correlates of impairments of platelet AANAT and ASMT activities in patients with ASD.

ADI-R, Autism Diagnostic Interview-Revised. RBS, Repetitive Behavior Scale. SRS, Social Responsiveness Scale. Intellectual disability is defined as verbal IQ and performance IQ<70.

For each biochemical parameter, patients with ASD were classified into normal and pathological group (high or low) on the basis of the threshold of the 10th percentiles of the control group. The two groups were compared using the Fisher's exact test for categorical items (n and % of individuals with diagnosis are indicated) and the Wilcoxon two-sample test for quantitative items (medians and quartiles are indicated). Because the number of individuals investigated displays mild variations across biochemical parameters and across clinical items, the total sample size (n) is indicated separately for each test.

	**patients with**	**low platelet AANAT activity**	**normal platelet AANAT activity**	***p***	**low platelet ASMT activity**	**normal platelet ASMT activity**	***p***
**ADI**	n	55	99		109	31	
B (social)	median [quartiles]	24 [16-27]	24 [18-27]	*0.87*	23 [17-26]	23 [14-28]	*0.64*
C (verbal comm.)	median [quartiles]	15 [10-18]	16 [13-18]	*0.39*	16 [13-19]	15 [9-17]	*0.19*
C (non verbal comm.)	median [quartiles]	12 [7-14]	12 [9-14]	*0.79*	12 [8-14]	10 [5-14]	*0.14*
D (repetitive behavior)	median [quartiles]	6 [4-9]	6 [4-8]	*0.73*	6 [4-8]	6 [4-7]	*0.30*
	n	35	43		56	20	
**RBS** (total score)	median [quartiles]	24 [10-33]	18 [12-34]	*0.47*	20 [9-33]	24 [13-36]	*0.57*
**SRS** (total score)	median [quartiles]	87 [74-99]	86 [67-97]	*0.46*	87 [72-99]	86 [68-96]	*0.69*
	n	64	97		109	42	
**Intellectual disability**	n (%)	32 (50 %)	51 (53 %)	*0.75*	54 (50 %)	19 (45 %)	*0.64*
	n	67	67		101	31	
**Insomnia** (self-report)	n (%)	31 (46%)	23 (34%)	*0.22*	46 (46%)	7 (23%)	***0.035***

Supplementary Table 2: biochemical and clinical data for families carrying rare AANAT and ASMT variants.

Families described in our previous study[12], or families for which no clinical or biochemical data were available, were not included in this table. Subjects carrying rare variants are indicated in grey. ASD probands are indicated in red. Normal/pathological categories for biochemical parameters were determined using the 95th or 5th percentile of the control group as a threshold, *i.e.* for AANAT activity: 2.9 pmol/10^9 platelets/30 min, for NAS: 44 nmol/10^9 platelets, for ASMT activity: 0.88 pmol/10^9 platelets/30 min and for melatonin: 0.07 nM.

GA generalized anxiety, MDD major depressive disorder, OCD/OCS obsessive-compulsive disorder/symptoms, PDAG panic disorder with agoraphobia, PDD-NOS Pervasive developmental disorder, not otherwise specified, SAD social anxiety disorder, TS Tourette Syndrome.

[a]ASMT M198R variant was predicted *in silico* as benign, but its functional impact was not determined in vitro.

Supplementary table 2. Biochemical and clinical data for families carrying rare AANAT and ASMT variants.

mutation	impact of mutation on protein function	family	status	genotype	Axis I diagnosis	sleep disorders	platelet AANAT activity (pmol/10^9 platelets/30 min)	platelet NAS (nmol/10^9 platelets)	platelet ASMT activity (pmol/10^9 platelets/30 min)	plasma melatonin (nM)
AANAT T3M	damaging	RD-154	father	*AANAT* T3M/+	-	ND	**3,9 (normal)**	22,0 (normal)	1,94 (normal)	0,27 (normal)
			mother	*AANAT* +/+	-	ND	**3,2 (normal)**	27,4 (normal)	0,83 (low)	0,07 (low)
			proband (M)	*AANAT* T3M/+	autism	ND	**3,7 (normal)**	26,8 (normal)	0,83 (low)	0,07 (low)
		RD-165	father	*AANAT* T3M/+<br>*ASMT* +/+	MDD	no sleep disorders	**3,3 (normal)**	12,3 (normal)	**0,74 (low)**	0,07 (low)
			mother	*AANAT* +/+<br>*ASMT* CNV/+	MDD	no sleep disorders	**3,6 (normal)**	9,9 (normal)	**1,86 (normal)**	0,22 (normal)
			proband (M)	*AANAT* T3M/+<br>*ASMT* CNV/+	Asperger	no sleep disorders	**6,3 (normal)**	2,5 (normal)	**3,36 (normal)**	0,39 (normal)
		001-022	proband (F)	*AANAT* T3M/+<br>*ASMT* CNV/+	autism, MDD, ADHD	phase delay, insomnia	**3,4 (normal)**	36,4 (normal)	**0,80 (low)**	0,06 (low)
AANAT A13S	benign	RD-185	father	*AANAT* A13S/+	-	ND	**4,8 (normal)**	24,0 (normal)	1,43 (normal)	0,17 (normal)
			mother	*AANAT* +/+	-	ND	**5,1 (normal)**	77,0 (high)	0,18 (low)	0,03 (low)
			proband (M)	*AANAT* A13S/+	autism	ND	**3,7 (normal)**	26,6 (normal)	0,68 (low)	0,07 (low)
AANAT R53C	damaging	RD-003	father	*AANAT* +/+	-	ND	**ND**	ND	ND	ND
			mother	*AANAT* R53C/+	-	ND	**ND**	ND	ND	ND
			proband (M)	*AANAT* R53C/+	autism	ND	**4,6 (normal)**	32,9 (normal)	0,46 (low)	0,05 (low)
AANAT A157V	benign	RD-133	father	*AANAT* +/+	OCD	ND	**3,6 (normal)**	32,9 (normal)	0,65 (low)	0,07 (low)
			mother	*AANAT* A157V/+	-	ND	**4,1 (normal)**	39,7 (normal)	0,55 (low)	0,06 (low)
			proband (M)	*AANAT* A157V/+	autism	ND	**3,7 (normal)**	34,1 (normal)	0,65 (low)	0,05 (low)
AANAT A163V	damaging	001-025	proband (M)	*AANAT* A163V/+	Asperger, MDD	phase delay, insomnia, nightmares	**5,0 (normal)**	33,4 (normal)	0,87 (low)	0,08 (normal)
		RD-228	proband (M)	*AANAT* A163V/+	Asperger	no sleep disorders	**3,8 (normal)**	48,3 (high)	0,18 (low)	0,02 (low)
ASMT L11F	damaging	RD-251	mother	*ASMT* +/+	-	ND	ND	ND	**ND**	ND
			father	*ASMT* L11F/+	-	ND	ND	ND	**ND**	ND
			proband (M)	*ASMT* L11F/+	PDD-NOS	no sleep disorders	3,6 (normal)	30,0 (normal)	**0,93 (normal)**	0,09 (normal)
ASMT N17K	damaging	RD-223	mother	*ASMT* N17K/+	MDD, SAD	insomnia	4,2 (normal)	40,2 (normal)	**0,73 (low)**	0,25 (normal)
			father	*ASMT* +/+	OCS	no sleep disorders	4,7 (normal)	35,6 (normal)	**1,05 (normal)**	0,17 (normal)
			sister	*ASMT* N17K/+	SAD	no sleep disorders	4,7 (normal)	42,2 (normal)	**0,99 (normal)**	0,08 (normal)
			proband (M)	*ASMT* N17K/+	autism, PDAG, OCD, ADHD, tics	phase delay, insomnia	2,3 (low)	46,4 (high)	**0,81 (low)**	0,1 (normal)

ASMT I269M	damaging	FRA-036	father	*ASMT* +/+	SAD, OCD	no sleep disorders	4,4 (normal)	41,7 (normal)	**0,47 (low)**	0,06 (low)
			mother	*ASMT* I269M/+	-	insomnia	4,3 (normal)	47,6 (high)	**0,85 (low)**	0,08 (normal)
			sister	*ASMT* +/+	MDD	no sleep disorders	5,1 (normal)	72,0 (high)	**0,27 (low)**	0,04 (low)
			proband (M)	*ASMT* I269M/+	autism, ADHD	no sleep disorders	3,6 (normal)	25,0 (normal)	**1,38 (normal)**	0,14 (normal)
			affected brother	*ASMT* +/+	autism	no sleep disorders	3,9 (normal)	34,0 (normal)	**0,86 (low)**	0,05 (low)
ASMT C273S	damaging	RD-112	mother	*ASMT* C273S/+	-	no sleep disorders	4,7 (normal)	37,0 (normal)	**0,86 (low)**	0,08 (normal)
			proband (M)	*ASMT* C273S/+	autism	no sleep disorders	3,7 (normal)	24,9 (normal)	**0,85 (low)**	0,04 (low)
ASMT M198R	benign[a]	RD-067	father	*ASMT* M198R/+	-	ND	ND	ND	**ND**	ND
			mother	*ASMT* +/+	-	ND	ND	ND	**ND**	ND
			proband (F)	*ASMT* M198R/+	autism	phase delay	ND	ND	**ND**	ND
ASMT CNV	ND	FRA-033	father	*ASMT* +/+	Tics	phase delay, insomnia	ND	ND	**ND**	ND
			mother	*ASMT* CNV/+	MDD	hypersomnia, nightmares	ND	ND	**ND**	ND
			proband (M)	*ASMT* CNV/+	autism, Tics, TS	insomnia, nightmares	ND	ND	**ND**	ND
			affected brother	*ASMT* CNV/+	Asperger, ADHD, Tics	phase delay	ND	ND	**ND**	ND
		RD-202	father	*ASMT* +/+	-	ND	ND	ND	**ND**	ND
			mother	*ASMT* CNV/+	-	ND	ND	ND	**ND**	ND
			proband (M)	*ASMT* CNV/+	autism	ND	0,9 (low)	33,2 (normal)	**0,53 (low)**	0,05 (normal)
		RD-180	father	*ASMT* +/+	-	ND	ND	ND	**ND**	ND
			mother	*ASMT* CNV/+	-	ND	ND	ND	**ND**	ND
			proband (F)	*ASMT* CNV/+	autism	ND	2,6 (low)	11,4 (normal)	**1,44 (normal)**	0,10 (normal)
		RD-284	father	*ASMT* CNV/+	-	somnambulism, nighmares	3,7 (normal)	24,7 (normal)	**1,58 (normal)**	0,16 (normal)
			mother	*ASMT* +/+	OCD, Tics, TS	no sleep disorders	5,6 (normal)	56 (high)	**0,29 (low)**	0,04 (low)
			proband (M)	*ASMT* CNV/+	autism	insomnia	3,0 (normal)	47,2 (high)	**0,85 (low)**	0,09 (normal)
		RD-281	mother	*ASMT* CNV/+	-	no sleep disorders	3,8 (normal)	31,5 (normal)	**0,65 (low)**	0,05 (low)
			proband (M)	*ASMT* CNV/+	autism	insomnia	1,9 (low)	39,6 (normal)	**0,49 (low)**	0,06 (low)
		RD-209	father	*ASMT* CNV/+	-	phase delay	2,1 (low)	37 (normal)	**0,86 (low)**	0,29 (normal)
			mother	*ASMT* CNV/+	GA	no sleep disorders	2,4 (low)	32,9 (normal)	**1,48 (normal)**	0,25 (normal)
			proband (M)	*ASMT* CNV/+	autism, OCD	nocturnal terror, nighmares	2,6 (low)	47,4 (high)	**0,66 (low)**	0,08 (normal)
			sister	*ASMT* CNV/+	-	no sleep disorders	2,5 (low)	43,2 (high)	**0,51 (low)**	0,20 (normal)
		001-018	father	*ASMT* +/+	-	insomnia	4,7 (normal)	37,0 (normal)	**0,69 (low)**	0,06 (low)
			mother	*ASMT* CNV/+	MDD	phase delay	4,7 (normal)	30,0 (normal)	**0,90 (low)**	0,10 (normal)
			proband (M)	*ASMT* CNV/+	autism	insomnia	3,1 (normal)	46,6 (high)	**0,26 (low)**	0,02 (low)
		001-024	proband (M)	*ASMT* CNV/+	Asperger, Tics, PDAG	no sleep disorders	3,3 (normal)	27,8 (normal)	**0,95 (normal)**	0,10 (normal)

Supplementary Table 3: characteristics of individual and control groups involved in the study of autopsy-derived tissues. Groups were compared using two-sample Wilcoxon test (age, PMI) or Fisher's exact test (gender, time of death). PMI: post-mortem interval.

		ASD patients	controls	p
**Ileum samples**				
**n**		13	11	
**Age** (years)	median [range]	15 [7-46]	15 [6-39]	0.81
**gender**	Male<br>Female	11<br>2	10<br>1	0.64
**PMI** (hours)	median [range]	19 [3-35]	14 [4-36]	0.32
**Pineal gland samples**				
**N**		9	22	
**Age** (years)	median [range]	39 [10-84]	36.5 [8-89]	0.98
**Gender**	Male<br>Female	8<br>1	20<br>2	0.86
**PMI** (hours)	median [range]	22 [16-35]	18.5 [5-31]	0.05
**time of death**	morning<br>afternoon<br>evening<br>night<br>unknown	0<br>3<br>4<br>2<br>0	3<br>6<br>4<br>8<br>1	0.42

Supplementary Table 4: primers and PCR conditions for exon amplification and sequencing.

<table>
<tr><th>gene</th><th>exon</th><th>Forward PCR primer</th><th>Reverse PCR primer</th><th>annealing temperature</th><th>elongation duration</th><th>Sequencing primer</th></tr>
<tr><td>ASMT</td><td>1B</td><td>GAGGCAGGAGAATCGCTTGAA</td><td>CAACAATGGAACGTGAGTGTG</td><td>56°C</td><td>1 min</td><td></td></tr>
<tr><td>ASMT</td><td>2</td><td>TGGTGCAATCTCATTTGACTCTG</td><td>GGGTTCATGCCATTCTCCTG</td><td>64,5°C</td><td>40 sec</td><td></td></tr>
<tr><td>ASMT</td><td>3</td><td>TCCAGCTGTACAAGGCAAGAG</td><td>CTCCTCCACTGCCACTTCAC</td><td>62°C</td><td>1 min</td><td>Forward</td></tr>
<tr><td>ASMT</td><td>4</td><td>GCCTGGGCTACAGAGCTGAAA</td><td>CTCCTGGGTTGTGCCATTTG</td><td>55°C</td><td>30 sec</td><td></td></tr>
<tr><td>ASMT</td><td>5</td><td>CCTGTGGGGTATAGCTCCGTTC</td><td>CGCACATGTCAAAGCATCAGA</td><td>63°C</td><td>30 sec</td><td></td></tr>
<tr><td>ASMT</td><td>7</td><td>TGGGTTGGACCCTTCATGAGT</td><td>GTGTTTCCGGGAGTGAGAGGA</td><td>64°C</td><td>30 sec</td><td></td></tr>
<tr><td>ASMT</td><td>8</td><td>AGCCTGGAAGACCTGGGAAAG</td><td>CCTGTGGGATGATTTCAGTGC</td><td>64°C</td><td>30 sec</td><td></td></tr>
<tr><td>ASMT</td><td>9</td><td>GGTGCCCTGACTGTCCTCTGA</td><td>CCATCAGCGTGGTCCTCAGTA</td><td>64°C</td><td>30 sec</td><td></td></tr>
<tr><td>AANAT</td><td>1</td><td rowspan="2">GCTACAAAAGAGGCCAGATACA</td><td rowspan="2">CAGTTCTGGAGGAAGAAATCC</td><td rowspan="2">58°C</td><td rowspan="2">1 min</td><td></td></tr>
<tr><td>AANAT</td><td>2</td><td>CAAGAAAGTGGGGGAAACAG</td></tr>
<tr><td>AANAT</td><td>3</td><td>GAGCACGTGTCAGCAGAAGT</td><td>CCGGTCTCAGGTACAGAGTTC</td><td>61°C</td><td>1 min</td><td></td></tr>
</table>

Supplementary Table 5: Mapping and genotyping of ASMT microduplication.

Primers and conditions for *ASMT* CNV breakpoint determination (GenomeWalker experiment)

The partial duplication of *ASMT* gene was first screened by MLPA as previously described[13]. Four independent DNA samples positive for MLPA screening were then subjected to a genome walker experiment for CNV mapping, using Genome Walker Universal kit (Clontech, USA) according to the manufacturer's instructions. Briefly, DNAs were submitted to digestion by restriction enzymes (EcoRV, PvuII, StuI, SspI; one reaction for each enzyme and each DNA), followed by ligation of adapters. Nested PCRs were performed on the ligation products with the provided adaptor primers, together with gene-specific primers.

PCR	first primer	nested primer
putative 5' end	GGATCATTTGAGGTCAGCAGTTTGAGA	TGGGCGACAGAAGGAGACTCCATCTCA
putative 3' end	GGAGCTCTGGCTAAGGAATGCATGTCT	GCAGCACTTCTCATTCCAGGAGGAAGAA

Primers and conditions for *ASMT* CNV genotyping

PCR products were sequenced to obtain the CNV breakpoint: the microduplication spans exon 1 to 7 and forms a tandem structure, with its breakpoint located 0.7 kb downstream of exon 7 and 4.4 kb upstream of exon 1 of *ASMT*. A specific genotyping test was designed, made of two PCR reactions performed simultaneously, one CNV-specific and one control PCR. The test was performed on 20-40 ng of genomic DNA using the Qiagen Multiplex PCR kit. The presence of PCR products was assessed by migration in 2% agarose gel.

PCR	Forward PCR primer	Reverse PCR primer	annealing temperature	elongation duration	amplicon size (bp)
CNV-specific	GTGGTGACAGATCTCGGCTCCCTTCAA	GTCTGGCAGGACGGTTTCAG	66°C	90 sec	406
control	TGGTGCAATCTCATTTGACTCTG	GGGTTCATGCCATTCTCCTG			552

Supplementary Figure 1: The measured serotonin-NAS-melatonin blood metabolites and the detail of the two phases of inclusion for the platelet ASMT data.

(a) whole blood serotonin, (**b**) platelet NAS content, and (**c**) plasma melatonin data previously published[9].

(**d**) platelet ASMT data previously published[14], (**e**) independent platelet ASMT data collected for this study.

The control groups were compared to other groups using the Wilcoxon two-sample test. Boxes indicate medians and quartiles.

Supplementary figure 1

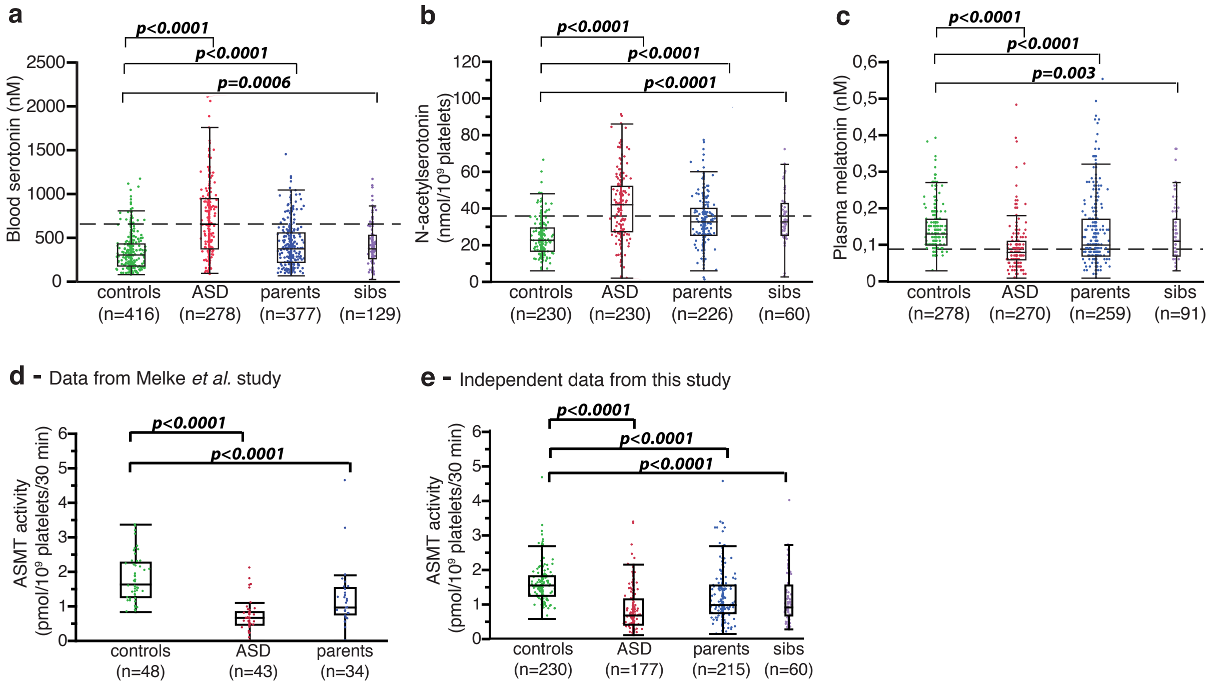

Supplementary Figure 2: Biochemical profiles of ASD patients.

a. All disturbed parameters (high blood serotonin, low platelet AANAT activity, high platelet NAS, low platelet ASMT activity, low plasma melatonin) were converted into percentiles for normalization (y axis). The median test was used to determine if the values observed are significantly above or below the 50th percentile of all patients (*$p < 0.05$; **$p < 0.01$; ***$p < 0.001$). Red lines, grey boxes and grey bars indicate medians, quartiles and ranges respectively.

b. Frequencies of the various biochemical profiles observed for patients with ASD.

Supplementary figure 2

a

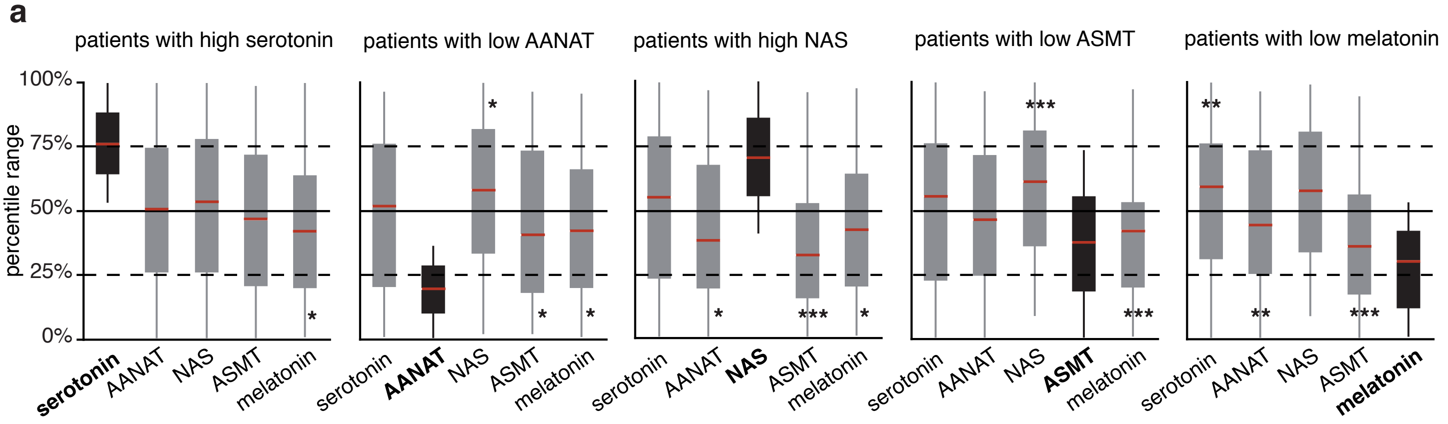

b

serotonin	AANAT	NAS	ASMT	melatonin	n (total=189)	%
high	low	high	low	low	22	11,6%
high	normal	high	low	low	22	11,6%
normal	low	high	low	low	16	8,5%
normal	normal	high	low	low	16	8,5%
normal	normal	normal	normal	normal	12	6,3%
high	normal	normal	low	low	12	6,3%
normal	normal	normal	low	low	10	5,3%
normal	low	normal	low	low	9	4,8%
normal	low	high	low	normal	8	4,2%
high	normal	high	low	normal	6	3,2%
normal	normal	normal	low	normal	6	3,2%
high	normal	normal	normal	normal	6	3,2%
normal	low	high	normal	normal	5	2,6%
normal	low	normal	normal	normal	5	2,6%
normal	normal	high	normal	normal	5	2,6%
high	low	high	low	normal	4	2,1%
normal	normal	high	low	normal	4	2,1%
high	low	normal	low	low	4	2,1%
high	low	normal	normal	low	4	2,1%
high	low	normal	normal	normal	4	2,1%
high	normal	high	normal	low	2	1,1%
high	normal	high	normal	normal	2	1,1%
high	normal	normal	low	normal	2	1,1%
normal	low	normal	normal	low	1	0,5%
high	low	high	normal	normal	1	0,5%
high	normal	normal	normal	low	1	0,5%

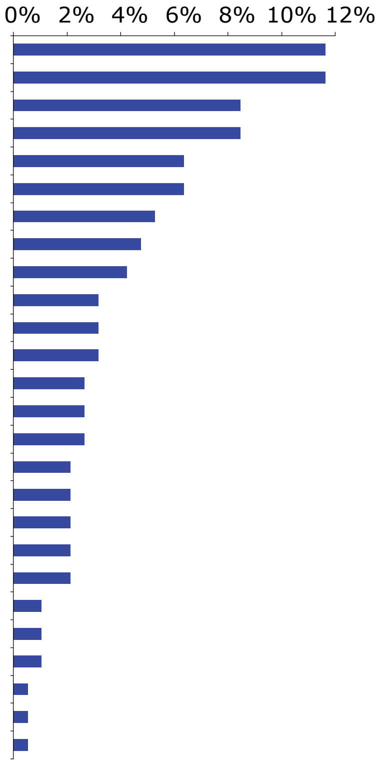

References

1 Constantino, J. N., Przybeck T., Friesen D. & Todd, R. D. Reciprocal social behavior in children with and without pervasive developmental disorders. *J. Dev. Behav. Pediatr.* **21**, 2–11 (2000).

2 Lord, C., Rutter, M. & Le Couteur, A. Autism Diagnostic Interview-Revised: a revised version of a diagnostic interview for caregivers of individuals with possible pervasive developmental disorders. *J. Autism Dev. Disord.* **24**, 659–685 (1994).

3 Lord, C. *et al.* The autism diagnostic observation schedule-generic: a standard measure of social and communication deficits associated with the spectrum of autism. *J. Autism Dev. Disord.* **30**, 205–223 (2000).

4 Lam, K. S. & Aman, M. G. The Repetitive Behavior Scale-Revised: independent validation in individuals with autism spectrum disorders. *J. Autism Dev. Disord.* **37**, 855–866 (2007).

5 Launay, J. M., Geoffroy, C., Costa, J. L. & Alouf, J. E. Purified -SH-activated toxins (streptolysin O, alveolysin): new tools for determination of platelet enzyme activities. *Thromb. Res.* **33**, 189-196 (1984).

6 Launay, J. M. & Alouf, J. E. Biochemical and ultrastructural study of the disruption of blood platelets by streptolysin O. *Biochim. Biophys. Acta* **556**, 278-291 (1979).

7 Namboodiri, M. A. A., Nakai, C. & Klein, D. C. Effects of selected treatments on stability and activity of pineal serotonin N-acetyltransferase. *J. Neurochem.* **33**, 807-810 (1979).

8 Axelrod, J., Wurtman, R. J. & Snyder, S. H. Control of hydroxyl-indole-O-methyltransferase activity in rat pineal gland by environmental lighting. *J. Biol. Chem.* **240**, 949-954 (1965).

9 Kirschner, M. B. *et al.* Haemolysis during sample preparation alters microRNA content of plasma. *PLoS One* **6**, e24145 (2011).

10 Devaux, Y. *et al.* Use of circulating microRNAs to diagnose acute myocardial infarction. *Clin. Chem.* **58**, 559-567 (2012).

11 Goubran-Botros, H. G. *et al.* Crystal structure and functional mapping of human ASMT, the last enzyme of the melatonin synthesis pathway. *J. Pineal Res.* **54**, 46–57 (2013).

12 Pagan, C. *et al.* The serotonin-N-acetylserotonin-melatonin pathway as a biomarker for autism spectrum disorders. *Transl. Psychiatry* **4**, e479 (2014).

13 Cai, G. *et al.* Multiplex ligation-dependent probe amplification for genetic screening in autism spectrum disorders: efficient identification of known microduplications and identification of a novel microduplication in ASMT. *BMC Med. Genomics* **1**, 50 (2008).

14 Melke, J. *et al.* Abnormal melatonin synthesis in autism spectrum disorders. *Mol. Psychiatry* **13**, 90–98 (2008).
